# Minocycline alleviates LPS-induced cognitive dysfunction in mice by inhibiting the NLRP3/caspase-1 pathway

**DOI:** 10.18632/aging.205528

**Published:** 2024-02-06

**Authors:** Fenfang Zhan, Yao Dong, Lanqian Zhou, Xiaozhong Li, Zheng Zhou, Guohai Xu

**Affiliations:** 1 Department of Anesthesiology, The Second Affiliated Hospital of Nanchang University, Nanchang, China; 2 Department of Cardiovascular Medicine, The Second Affiliated Hospital of Nanchang University, Nanchang, China; 3 Jiangxi Key Laboratory of Molecular Medicine, The Second Affiliated Hospital of Nanchang University, Nanchang, China

**Keywords:** minocycline, lipopolysaccharide, NOD-like receptor protein-3, caspase-1, cognitive dysfunction

## Abstract

Background: Growing experimental evidence indicates that cognitive impairment is linked to neuroinflammation. Minocycline (MINO), an antibiotic known for its anti-inflammatory, has shown promise in alleviating cognitive impairment. Nonetheless, the exact mechanism through which MINO improves cognitive impairment is not yet understood.

Methods: A neuroinflammatory model was establish by utilizing lipopolysaccharide. The assessment of mice's cognitive and learning abilities was conducted through the MWM and Y-maze tests. The evaluation of hippocampal neuronal injury and microglial activation were achieved by performing HE staining and IHC, respectively. To evaluate BV2 cell viability and apoptosis, the CCK-8 and Hoechst 33342/PI staining assays were employed. In order to assess the protein and RNA expression levels of NLRP3, caspase-1, IL-1β, IL-18, Iba-1, and Bcl2/Bax, WB and RT-qPCR were utilized. Additionally, the inhibitory effect of MINO on apoptosis by targeting the NLRP3/caspase-1 pathway was investigated using Nigericin.

Results: MINO was effective in reducing the time it took for mice to escape from the test, increasing the number of platforms they crossed, and mitigating damage to the hippocampus while also suppressing microglial activation and the expression of Iba-1 in a neuroinflammatory model caused by LPS. Furthermore, MINO improved the viability of BV2 cell and reduced apoptosis. It also had the effect of reducing the expression levels of NLRP3/Caspase-1, IL-1β, IL-18, and BAX, while upregulating the expression of Bcl2. Additionally, MINO was found to downregulate the NLRP3 expression, which is specifically activated by nigericin.

Conclusion: The protective effect of MINO relies on the crucial involvement of the NLRP3/caspase-1 pathway.

## INTRODUCTION

Cognitive dysfunction is a crucial feature observed in numerous neurodegenerative disorders, like Parkinson’s disease (PD), Alzheimer’s disease (AD), and Huntington’s disease (HD). Research conducted in the past has revealed the substantial role played by neuroinflammation in contributing to impairments in cognitive functioning [1, 2]. Lipopolysaccharide (LPS) is a significant Toll-like receptor 4 (TLR4) ligand found Gram-negative bacteria, commonly employed to develop models of inflammation. According to research, injecting LPS into the lateral ventricle of mice has been found to increase the presence of inflammatory factors in the hippocampus, resulting in cognitive dysfunction [3, 4]. The growing body of evidence indicates that cognitive dysfunction can be improved through the use of anti-inflammatory treatments [5, 6]. Hence, the role of anti-inflammatory therapy in alleviating cognitive impairment is of utmost importance.

MINO, an antibiotic that is lipid-soluble, exhibits anti-inflammatory, anti-apoptotic, and antioxidant properties. Its neuroprotective effects within the central nervous system (CNS) are facilitated by the blood-brain barrier (BBB). Research findings have indicated that MINO possess a safeguarding influence on central nervous system (CNS) disorders, namely depression [7], ischemic stroke [8], AD [9], and PD [10]. Thus far, evidence has demonstrated that MINO mitigates cognitive dysfunction by diminishing inflammation and apoptosis [11, 12]. Nonetheless, the precise mechanism through which MINO exerts its neuroprotective properties still eludes researchers.

NOD-like receptor protein-3 (NLRP3) belongs to a family known as NOD-like receptors (NLRs), which consists of nucleotide-binding domains (NBD) and leucine-rich repeat (LRR) containing proteins [13]. Activation of NLRP3 leads to the activation of apoptosis-associated speck-like protein (ASC) and pro-caspase-1, resulting in the release and maturation of IL-1β and IL-18. This process contributes to the development of various diseases. Accumulating evidence suggests that NLRP3 plays a crucial role in the onset and progression of diseases affecting the CNS. Conditions such as traumatic encephalopathy (TBI) [14, 15], ischemic stroke [16], and hemorrhagic stroke [17] are associated with significantly increased expression of NLRP3. Inhibiting NLRP3 expression has shown promise ameliorating the development of these diseases. Furthermore, recent research has revealed that the activation of NLRP3 is strongly linked to the advancement of neurodegenerative disorders, like AD and PD [18–20]. Therefore, it is crucial to identify drugs that can inhibit NLRP3 activation. Recent studies have highlighted the effectiveness of MINO in inhibiting NLRP3 activation and ameliorating neuroinflammation. Su et al.’s study demonstrated that MINO can mitigate neuroinflammation caused by chronic cerebral hypoperfusion by inhibiting the activation of the NLRP3 inflammasome [21]. Additionally, researchers have observed that exposure to lead increase NLRP3 expression. However, MINO treatment partially reverses this effect, suggesting that MINO intervention could help alleviate lead-induced neurotoxicity [22]. Nevertheless, it remains uncertain whether MINO can inhibit the NLRP3/caspase-1 pathway, a neuroinflammatory marker, to enhance cognitive dysfunction induced by LPS. Thus, we formulated a hypothesis that MINO could improve cognitive function by inhibiting the NLRP3/caspase-1 pathway. To validate our hypothesis, we conducted both *in vivo* and *in vitro* experiments to explore the therapeutic effects of MINO on neuroinflammation.

## MATERIALS AND METHODS

### Animals and treatment

This investigation involved the utilization of 6-to 7- week-old C57BL/6J mice, which were purchased from Skobis Company (Henan, China). The mice were then placed and sustained within the Animal House of Nanchang University. A controlled environment was maintained within the facility, ensuring a balanced light/dark schedule of 12-hours (lights on from 8:00 a.m. to 8:00 p.m.). The mice were accommodated in polystyrene cages and provided unrestricted access to water. The Animal Care and Use Committee of the Second Affiliated Hospital of Nanchang University granted approval for all *in vivo* experiments conducted in this research (approval number: A20221031119). The principles outlined in the institutional guidelines were strictly adhered to throughout the course of this study.

A total of 36 C57BL/6J mice were divided into 3 groups (with 12 mice in each group), namely the control group, the LPS group, and the LPS+MINO group. For the LPS group, the mice were intracerebroventricularly injected with LPS (Escherichia coli 055:B5; 5 μg dissolved in 2 μl of normal saline) [23]. The control group, on the other hand, received an equivalent amount of saline. In the MINO group, the mice were pre-treated with minocycline (Med Chem Express, HY-17412, USA; 100 μg/kg (40 μg) dissolved in 2 μl of normal saline) one hour prior to the administration of LPS, consistent with a previous study [24]. Six mice from each group then underwent the Morris water maze (MWM) and Y-maze tests after the LPS injection. 24 hours after the LPS injection, the remaining mice were sacrificed for subsequent experiments, including western blotting, H&E staining, immunohistochemistry, immunofluorescence, and quantitative real-time polymerase chain reaction (qRT-PCR).

### Behavioral tests

The assessment of spatial learning and memory was conducted using the Morris water maze (MWM), which incorporated a video-tracking system manufactured by Shanghai Xinruan (XR-XM101, China). As previously described [25, 26], the MWM setup comprised of a round tank measuring 120 cm in diameter and 50 cm in height, a platform with a diameter of 10 cm, and a camera-based analysis system. To ensure accurate results, the round tank was divided into four quadrants, with the platform positioned 1 cm beneath the water surface. The MWM evaluation consisted of two main components: acquisition training and probe trials. In the acquisition training phase, mice were placed in the water from different quadrants, allowing them a maximum of 60 seconds per day to locate the platform. This procedure was repeated over five consecutive days, with the escape latency being recorded. In instances where mice failed to find the platform within the allotted time, they were gently guided to it using a stick and allowed to remain on the platform for 15 seconds. The subsequent day marked the commencement of the probe trials. During these trials, the platform was removed, and the mice were released into the pool from the opposite quadrant of the platform. The number of times each mouse crossed the platform within a 60-second window was recorded. After the completion of the probe trials, the mice were administered either LPS or saline into the lateral ventricle. Twenty-four hours subsequent to the LPS injection, the mice began repeating the acquisition training trials and probe trials. Both the escape latency and the number of platform crossings were documented.

After conducting the MWM test, we proceeded to the Y-maze test, a widely accepted technique used to evaluate short-term spatial memory and learning [27, 28]. The Y-maze test consisted of three identical arms positioned at an angle of 120°, each arm measuring 30 × 15 × 6 cm. These arms were designated as the initial arm, alternate arm, and new arm. The experiment comprised two phases, with a 2-hour interval between them. In the first phase, the new arm remained closed, allowing the animal to freely explore the initial arm for a duration of 3 minutes. Subsequently, in the second phase, which mirrored the first phase, the new arm was opened. We accurately documented the number of entries and the duration of the animal’s presence in the new arm. Following each trial, we thoroughly cleansed the apparatus with a solution of 5% acetic acid to eliminate any residual odors.

### Hematoxylin/Eosin (H&E) staining, immunohistochemistry (IHC), and immunofluorescence (IF)

Following the isolation of mice, the brain tissue was promptly fixed using a 4% formaldehyde solution. Paraffin embedding was then carried out, and the brain tissue was sliced into sections with a thickness of 5μm. The sections were then used for different procedures such as H&E staining, immunofluorescence, or immunohistochemistry. For the H&E staining of the 5 μm sections, a standard protocol for H&E staining was employed. In the case of immunofluorescence and immunohistochemistry, the sections underwent heat-induced antigen retrieval using a sodium citrate buffer (0.01 M, pH = 6.0) by boiling for 10 minutes. Subsequently, the sections were incubated with goat serum for an hour, followed by overnight incubation at 4°C with the primary antibodies mentioned below: anti-Iba-1 (1:800, #17198, CST), anti-NLRP3 (1:200, #GB114320-100, Servicebio, Wuhan, China), and anti-caspase-1 (1:200, #GB11383-100, Servicebio, Wuhan, China). Sections incubated with anti-Iba-1 antibodies were subjected to further incubation with an HRP-polymer secondary antibody for 20 minutes. Afterwards, they were treated with DAB solution for 10 minutes to visualize the levels of Iba-1 protein using an optical microscope. The sections incubated with the other two antibodies were processed using a TSA fluorescent double-staining kit and counterstained with DAPI. Finally, representative images were captured using a fluorescence microscope.

### Cell culture and treatment

The BV-2 cell line derived from mouse microglia was obtained from Procell, a company based in Wuhan, China. The cells were cultured in modified Eagle’s medium (DMEM) supplied by Procell, Wuhan, China, which contained 10% fetal bovine serum (FBS; Procell, Wuhan, China) and 1% penicillin-streptomycin (P/S; Procell, Wuhan, China). The culture was maintained at a temperature of 37°C in a humidified atmosphere containing 95% O_2_ and 5% CO_2_. To induce inflammation, the cells were plated onto six-well plates and treated with 1 μg/ml of LPS for a period of 24 hours [29, 30]. Minocycline at various concentrations was added to the BV2 cells 2 hours prior to LPS treatment [29]. In select experiments, the cells were first exposed to nigericin (10 μM) for 1 hour before MINO administration [31]. Following stimulation, the cells were collected for subsequent analysis.

### Cell viability assay

In accordance with scientific methodologies, BV2 cells were planted onto 96-well plates (Corning, USA) at a density of 2 × 10^4^ cells per well in 100 μL of medium containing 10% FBS. Following that, the plates were incubated within a 37°C environment with a 5% CO_2_ atmosphere for a duration of 24 hours. Sequentially, the BV2 cells were subjected to minocycline (ranging from 0 to 100 μM) alongside LPS for a period of 24 hours. Following this treatment, the Cell Counting Kit-8 (#C0038, Beyotime, Shanghai, China) solution was introduced to the 96-well plates containing the BV2 cells for a total of 1 hour. A microplate reader was employed to record the absorbance at 450 nm.

### Cell apoptosis analysis

Cell apoptosis in the different treatment groups was analyzed using the Hoechst 33342/PI (propidium iodide) double stain kit provided by Solar Bio (Wuhan, China). Following the manufacturer’s instructions, the Petri dishes were sequentially treated with Hoechst and PI staining solutions. The observed cell apoptosis was analyzed using the ImageJ software with the aid of a fluorescence microscope from Olympus (Tokyo, Japan).

### qRT-PCR

RNA was obtained from hippocampal tissues utilizing TRIzol reagent (Tian Gen, Beijing, China). The extracted RNA underwent reverse transcription to form cDNA using the Easy Script^®^ One-Step gDNA Removal and cDNA Synthesis Super Mix (Trans Gen, Beijing, China) on an ABI Verity PCR (Applied Biosystems, USA) instrument. Subsequently, the cDNA was amplified using Perfect Start^®^ Green qPCR Super Mix (Trans Gen, Beijing, China) according to the manufacturer’s instructions. The primer sequences for mRNAs can be found in Table 1. Each trial was performed thrice, and the Ct value was normalized to that of GAPDH for assessing relative expression. The relative mRNA expression was determined using the 2^−ΔΔCt^ method [32].

**Table 1:** Primer sequences of RT-qPCR.

**Gene**	**Primer sequences (5′–3′)**	**Product size(bp)**
NLRP3	F: GGAGTTCTTCGCTGCTATGTACTA	110
	R: GGACCTTCACGTCTCGGTTC	
Caspase-1	F: CAAACATTACTGCTATGGACAAGGC	85
	R: GTGATAAAGATTTGGCTTGCCTG	
IL-1β	F: TGGGAAACAACAGTGGTCAGG	83
	R: ATTAGAAACAGTCCAGCCCATACTT	
IL-18	F: GAAGGACACTTTCTTGCTTGCC	127
	R: TCCCCACCTAACTTTGATGTAAGTT	
GAPDH	F: GAACGGGAAGCTCACTGG	118
	R: GCCTGCTTCACCACCTTCT	

### Western blotting analysis

To perform the plagiarism check, some modifications can be made to the provided text. Here is the modified version: RIPA lysate (Solar Bio, Beijing, China) was used to separate hippocampal tissue and BV2 cells. The separation process was conducted on ice for a duration of 30 minutes, followed by ultracentrifugation at 4°C. The total protein concentration was determined using a BCA protein assay kit (Tian Gen, Beijing, China). Subsequently, the proteins were loaded onto a sodium dodecyl sulfate-polyacrylamide gel electrophoresis (SDS-PAGE) and transferred onto polyvinylidene difluoride (PVDF) membranes. At room temperature, the membranes were blocked for 1 hour with 5% skim milk. For the primary antibody incubation, the following antibodies were used at 4°C overnight: anti-NLRP3 (1:1000, ab263899, Abcam, USA), anti-caspase-1 (1:500, ab179515, Abcam, USA), anti-IL-1β (1:500, ab234437, Abcam, USA), anti-IL-18 (1:500, ab243091, Abcam, USA), and anti-GAPDH (1:2000, 10494-1-AP, Protein Tech, Wuhan, China). The membrane was then incubated with a horseradish peroxidase-labeled goat anti-rabbit or goat anti-mouse secondary antibody (1:5000, Solar Bio) at 37°C for 1 hour. Finally, chemiluminescence was utilized for membrane exposure, and the bands of immunoreactive substances were analyzed using Image Lab software (version 3.0).

### Statistical analysis

Data are expressed as mean ± standard deviations (SD). GraphPad Prism (version 8.0) was used to conduct statistical analyses. For comparisons between the two groups, a two-tailed Student’s *t*-test employed. When comparing more than two groups, and assuming normal distribution with uniform variance, one-way or two-way ANOVA with Tukey’s multiple comparison test was used as a post hoc analysis if there were significant overall group differences. Alternatively, if the data did not follow a normal distribution or exhibited uneven variance, but with differing overall group means, the Kruskal-Wallis test with Dunnett’s Multiple Comparison was utilized as a post hoc test. Statistical significance was set at *p* < 0.05.

### Data availability

Data will be made available on request.

## RESULTS

### Minocycline alleviated LPS-induced cognitive dysfunction in mice

**Figure 1:**
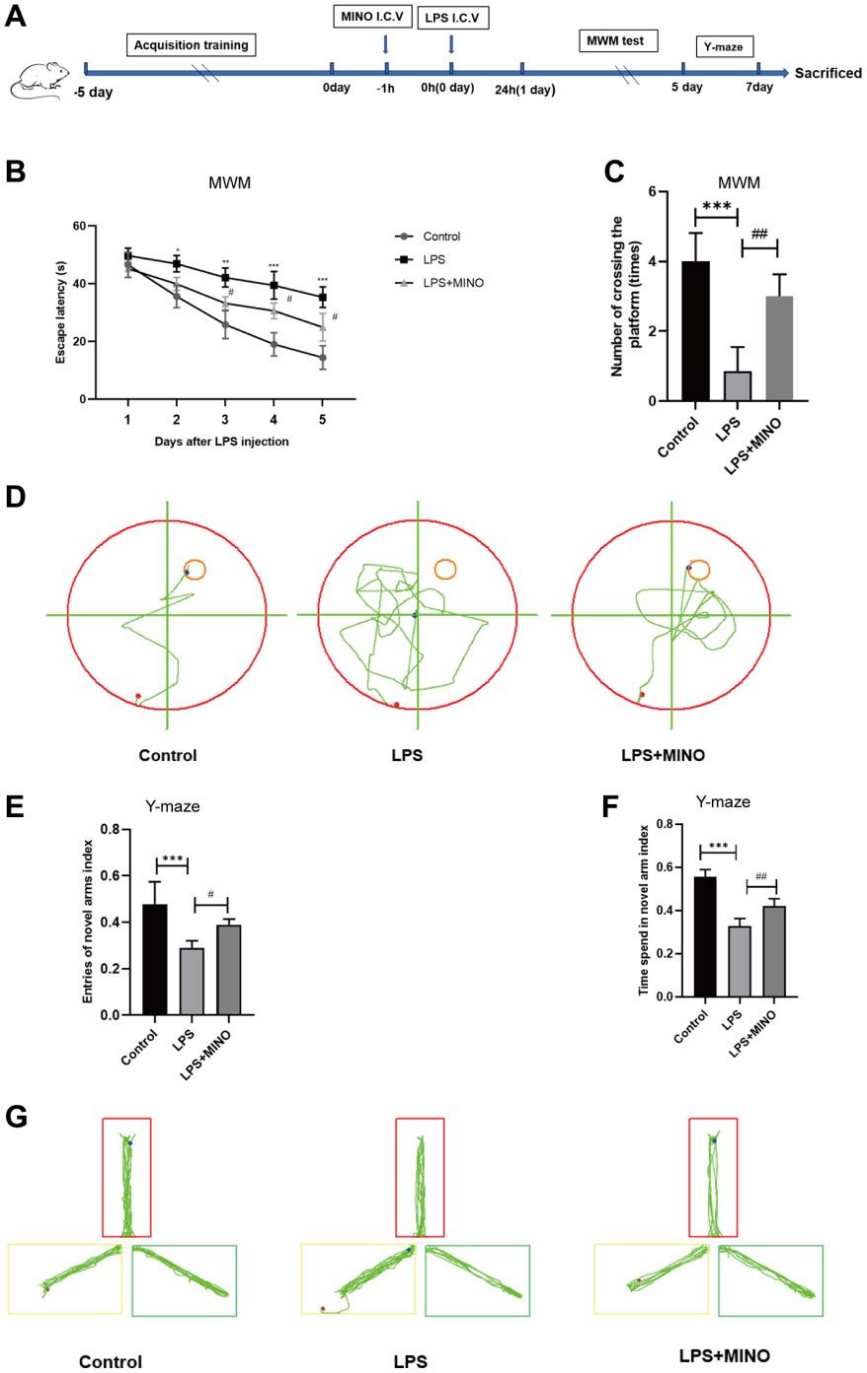
**Minocycline improved LPS-induced learning and memory impairment in mice.** (**A**) Schematic representation of the animal experiment. (**B**) The mean escape latency to the visible platform of the MWM test. (**C**) Numbers of platform location crossings during the probe trial test of MWM test. (**D**) A representative trajectory diagram of the MWM test. (**E**) Ratio of entering the novel arm. (**F**) Ratio of spending time in the novel arm. (**G**) A representative trajectory diagram of the Y-maze. Data were presented as mean ± SD; *n* = 6 mice per group. ^*^P < 0.05, ^**^P < 0.01, ^***^P < 0.001 vs. Control group; ^#^*P* < 0.05, ^##^*P* < 0.01, ^###^*P* < 0.001 vs. LPS group.

In order to evaluate the cognitive abilities related to learning and memory, we conducted a series of tests on mice, namely the Morris water maze (MWM) and Y-maze test. The step-by-step process of these experiments can be found in Figure 1A. The findings from the MWM escape experiment indicated a significant prolongation in escape latency in the LPS-treated group compared to the control group, upon 1 day of LPS treatment. Interestingly, the group that received both LPS and MINO exhibited a noticeable reduction in escape latency after 2 days of LPS treatment (Figure 1B). The MWM spatial exploration experiment revealed a remarkable decrease in the number of platform crossings in the LPS-treated group as opposed to the control group. Conversely, the LPS+MINO group displayed a remarkable increase in the number of platform crossings compared to the LPS group (Figure 1C). The trajectory of water maze activity for each experimental group was meticulously recorded and can be observed in Figure 1D.

To provide further clarification, we conducted the Y-maze test to evaluate the immediate spatial working memory of mice. The results depicted in Figure 1E–1G indicate that the mice in the LPS group had a decreased frequency of entering the novel arm and a lower entry index when compared to the control mice. In contrast, the mice in the LPS + MINO group demonstrated a higher frequency of entering the novel arm and a greater entry index compared to the control mice. These findings suggest that MINO may have the ability to improve the cognitive impairment induced by LPS.

### Minocycline alleviated LPS-induced hippocampal injury and apoptosis in mice

**Figure 2:**
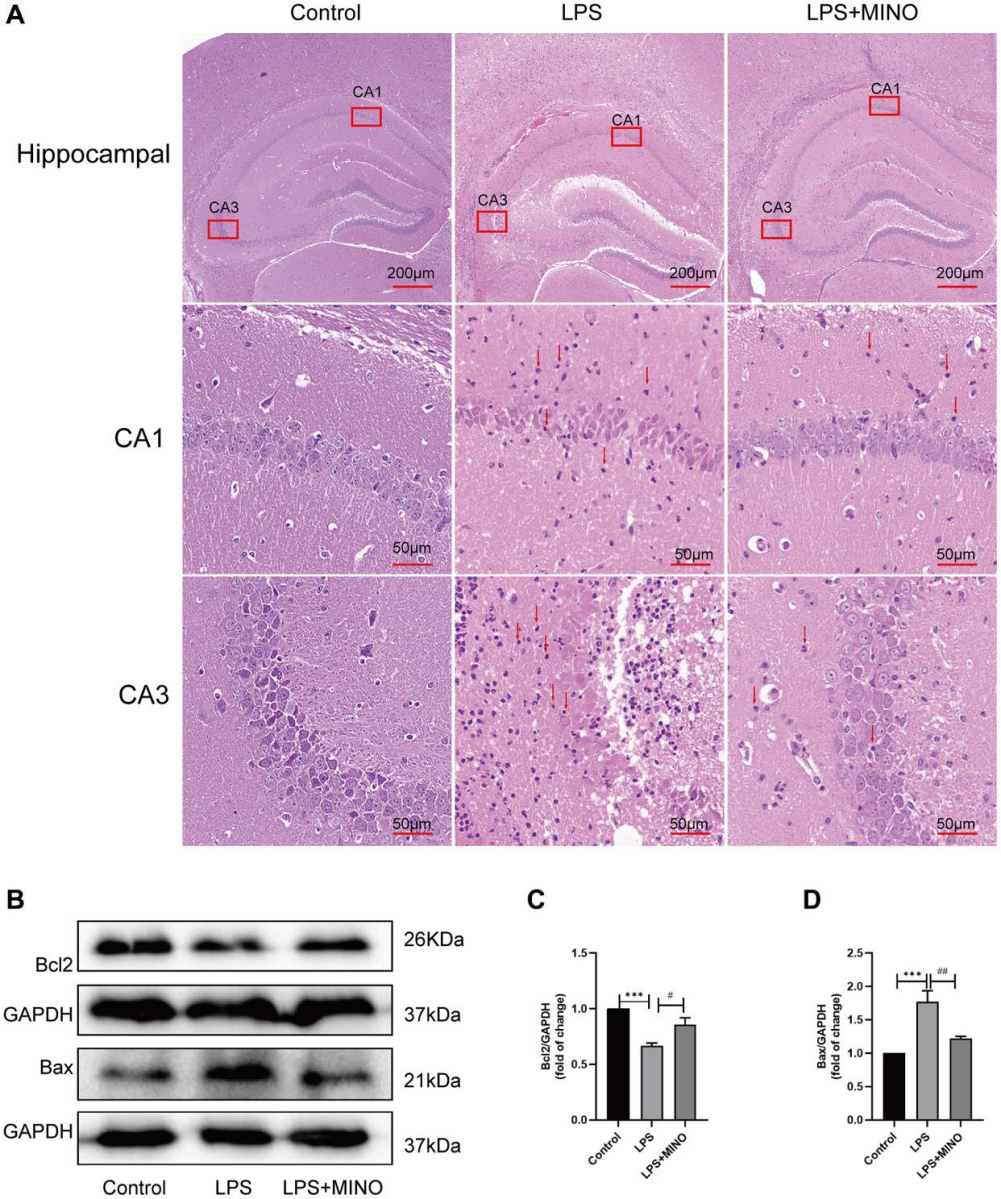
**Minocycline alleviated LPS-induced hippocampal injury and apoptosis.** (**A**) Hippocampal tissues were stained with H&E solution to elucidate pathological alterations. The red arrows point to the inflammatory cells. (**B**–**D**) The expression levels of Bax and Bcl-2 in hippocampal tissues were detected by Western blot assay. Data were presented as mean ± SD, *n* = 5–6 mice per group; ^*^P < 0.05, ^**^P < 0.01, ^***^P < 0.001 vs. Control group; ^#^*P* < 0.05, ^##^*P* < 0.01, ^###^*P* < 0.001 vs. LPS group.

In this study, we employed H&E staining (Figure 2A) to examine the tissue structure and neuronal morphology of the hippocampus. The findings demonstrated that the hippocampus of mice in the LPS group experienced a notable influx of inflammatory cells compared to the control group. Furthermore, neuronal cells exhibited distinct characteristics, including shrinkage, plasma membrane blebbing, significant nuclear condensation, and a disruption in their arrangement. Nevertheless, the administration of minocycline in the LPS + MINO group demonstrated a remarkable decrease in the infiltration of inflammatory cells when compared to the LPS group. Moreover, minocycline treatment effectively mitigated neuronal cell shrinkage, plasma membrane blebbing, and nuclear condensations.

Additionally, western blotting was used to identify proteins associated with apoptosis (Figure 2B–2D). The findings revealed a significant decrease in the level of Bcl-2, while the level of Bax exhibited a remarkable increase in the LPS group compared to the control group. Conversely, in the LPS+MINO group, the Bcl-2 level showed a considerable increase, while the Bax level displayed a remarkable decrease when compared to the LPS group. These outcomes suggest that MINO treatment effectively mitigated hippocampal injury and apoptosis induced by LPS.

### Minocycline inhibited microglia activation in LPS-induced mice’s hippocampus

**Figure 3:**
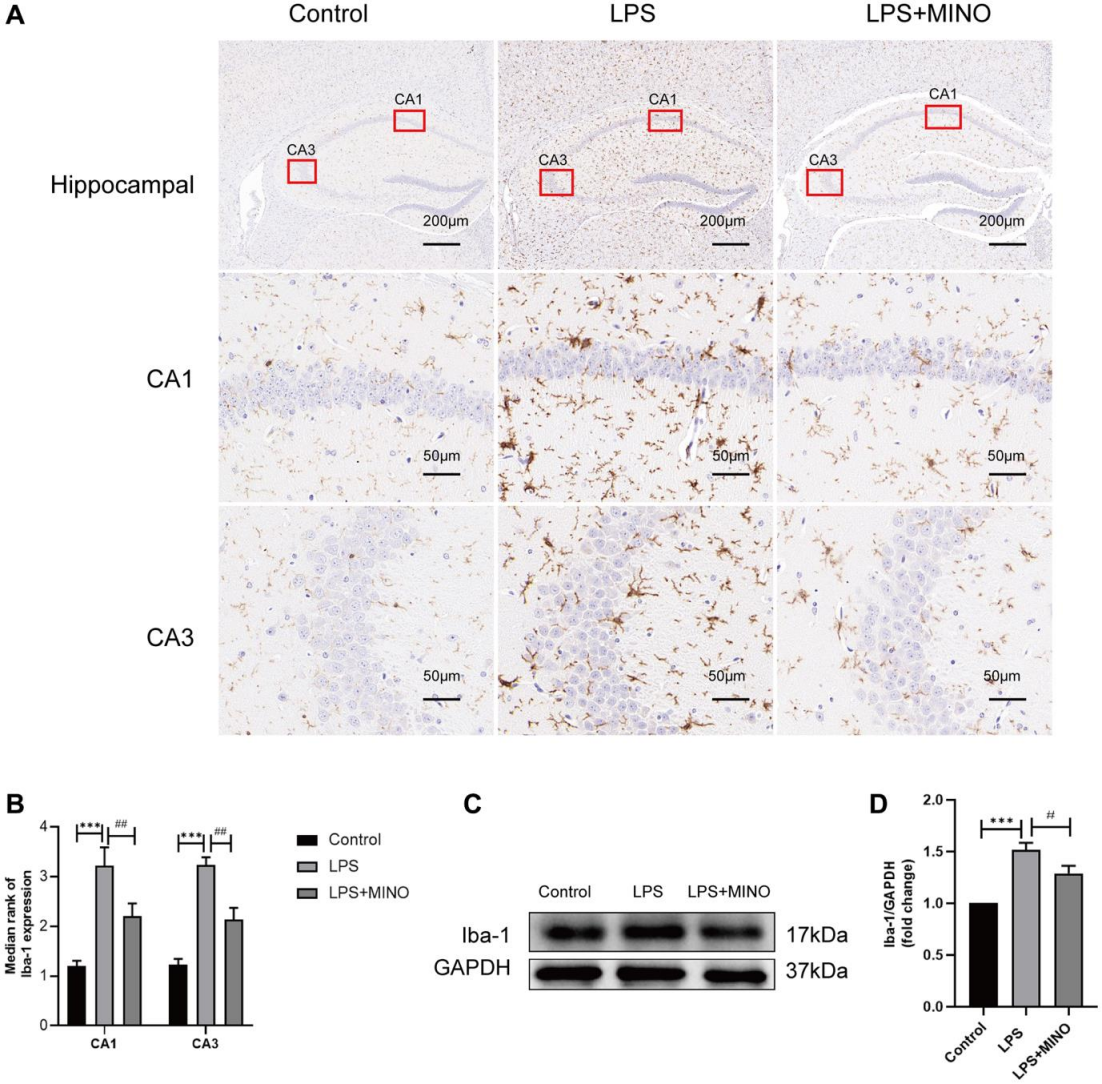
**Minocycline inhibited LPS-induced microglial activation in the mice hippocampus.** (**A**, **B**) An IHC staining assay was performed to detect the Iba-1 level in hippocampal tissues. (**C**, **D**) The expression level of Iba-1 was elucidated by a Western blot assay. Data were presented as mean ± SD, *n* = 5–6 mice per group; ^*^P < 0.05, ^**^P < 0.01, ^***^P < 0.001 vs. Control group; ^#^*P* < 0.05, ^##^*P* < 0.01, ^###^*P* < 0.001 vs. LPS group.

Iba-1, an essential indicator of microglial activation, exhibits an inclination towards increased expression during microglial activation [33, 34]. In our investigation, we evaluated Iba-1 levels in the hippocampus through both immunohistochemistry and western blotting techniques. Immunohistochemistry analysis revealed a substantial presence of Iba-1-positive cells in the LPS group compared to the control group. Notably, after pretreatment with minocycline, the LPS + MINO group exhibited a significant reduction in Iba-1-positive cells, as opposed to the LPS group (as displayed in Figure 3A, 3B). Furthermore, western blotting outcomes demonstrated a noteworthy elevation in the levels of Iba-1 in the LPS group when compared to the control group. Conversely, the LPS+MINO group exhibited a remarkable decrease in Iba-1 levels compared to the LPS group (as depicted in Figure 3C, 3D). These findings strongly imply that MINO effectively quelled LPS-induced microglial activation in the hippocampus of mice.

### Minocycline inhibited the NLRP3/Casapase-1 pathway activation in the hippocampus of LPS-treated mice

Prior research has established that the injection of LPS triggers activation of the signaling pathway NLRP3/caspase-1 in the hippocampus. To evaluate the impact of minocycline on the NLRP3/caspase-1 pathway, the researchers employed techniques such as western blotting, immunofluorescence staining, and qRT-PCR.

**Figure 4:**
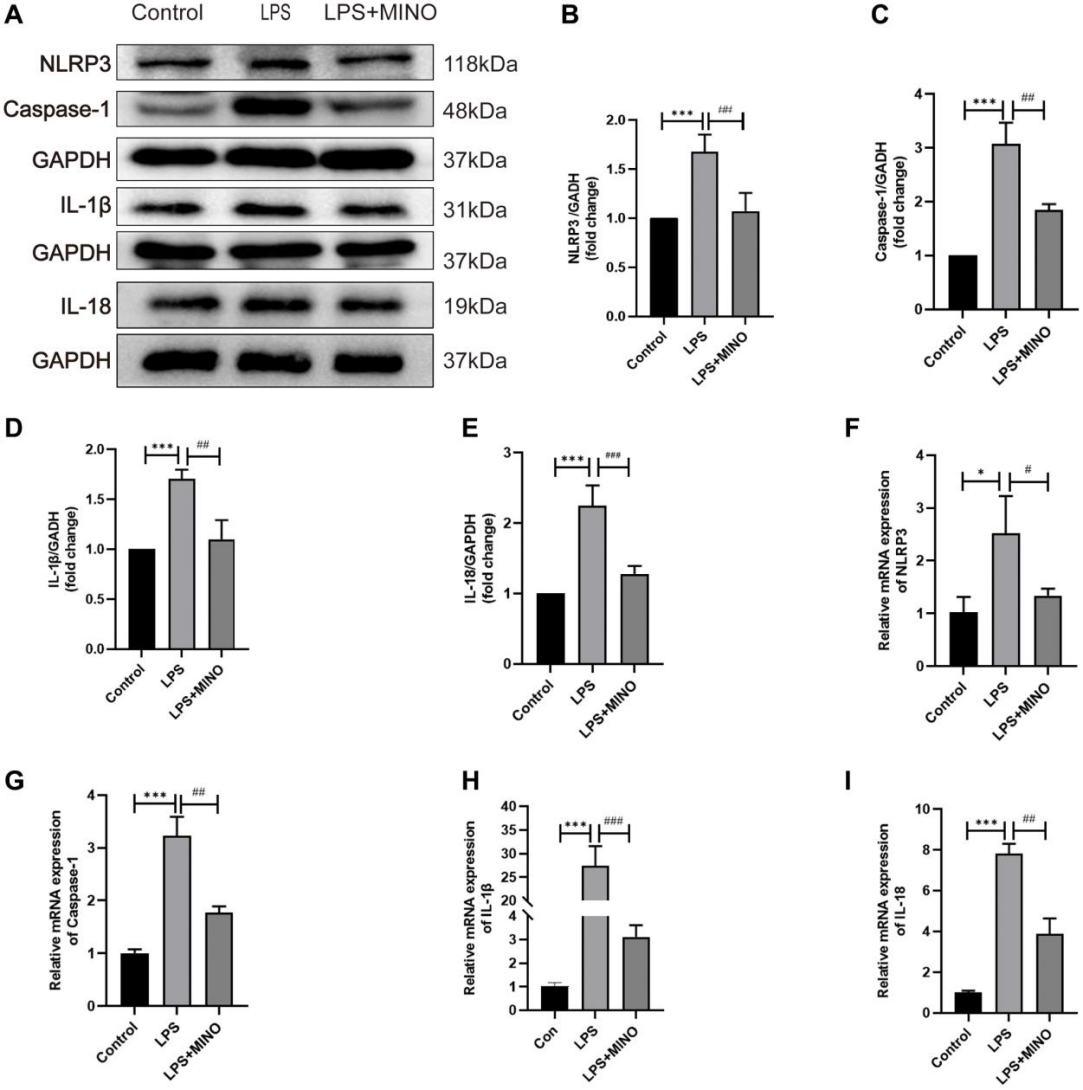
**Minocycline inhibited the NLRP3/Casapase-1 pathway in the hippocampus of LPS-treated mice.** (**A**) Representative Western blot bands of NLRP3, Caspase-1, IL-1β, and IL-18, and (**B**–**E**) their statistical graphs. (**F**–**I**) The mRNA expression of NLRP3, caspase-1, IL-1β, and IL-18. Data were presented as mean ± SD, *n* = 5–6 mice per group; ^*^P < 0.05, ^**^P < 0.01, ^***^P < 0.001 vs. control group; ^#^*P* < 0.05, ^##^*P* < 0.01, ^###^*P* < 0.001 vs. LPS group.

The results of the western blotting analysis showed that there was a notable increase in the protein levels of NLRP3, caspase-1, IL-1β, and IL-18 in the LPS group compared to the control group. However, pretreatment with minocycline effectively reduced the expression of these proteins in the LPS + MINO group (Figure 4A–4E). This suggests that minocycline has the potential to inhibit NLRP3-mediated inflammation.

*In vivo*, the immunofluorescence staining results were in agreement with the western blotting analysis for NLRP3 and caspase-1 (Supplementary Figure 1A–1C). Furthermore, the qRT-PCR analysis indicated that the mRNA levels of NLRP3, caspase-1, IL-1β, and IL-18 were markedly increased in the LPS group when compared to the control group. Conversely, in the LPS+MINO group, the mRNA levels of these molecules were significantly decreased in comparison to the LPS group (Figure 4F–4I). These findings provide evidence that the administration of MINO suppresses the activation of the NLRP3/caspase-1 pathway induced by LPS.

### Effects of minocycline on LPS-induced BV2 cell viability and apoptosis

**Figure 5:**
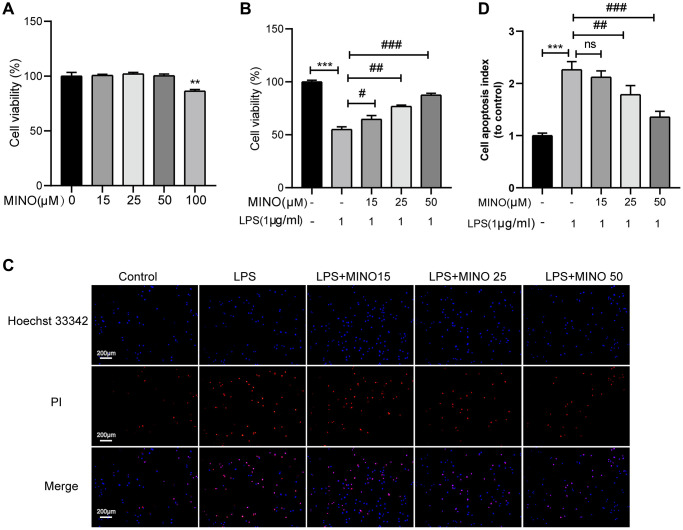
**The effects of minocycline on LPS-induced BV2 cell viability and apoptosis.** (**A**) Cells were treated with 0–100 μM minocycline for 24 h, and cell viability was detected by the CCK-8 assay. (**B**) Minocycline improved cell viability in LPS-treated BV2 cells (*n* = 3). (**C**) The apoptosis of BV2 cells was detected by the Hoechst 33,342/PI staining assay. (**D**) Quantitative analysis of BV2 cell apoptosis. Data were presented as mean ± SD; *n* = 4; ns: statistically non-significant; ^*^P < 0.05, ^**^P < 0.01, ^***^P <0.001 vs. control group; ^#^*P* < 0.05, ^##^*P* < 0.01, ^###^*P* < 0.001 vs. LPS group.

**Figure 6:**
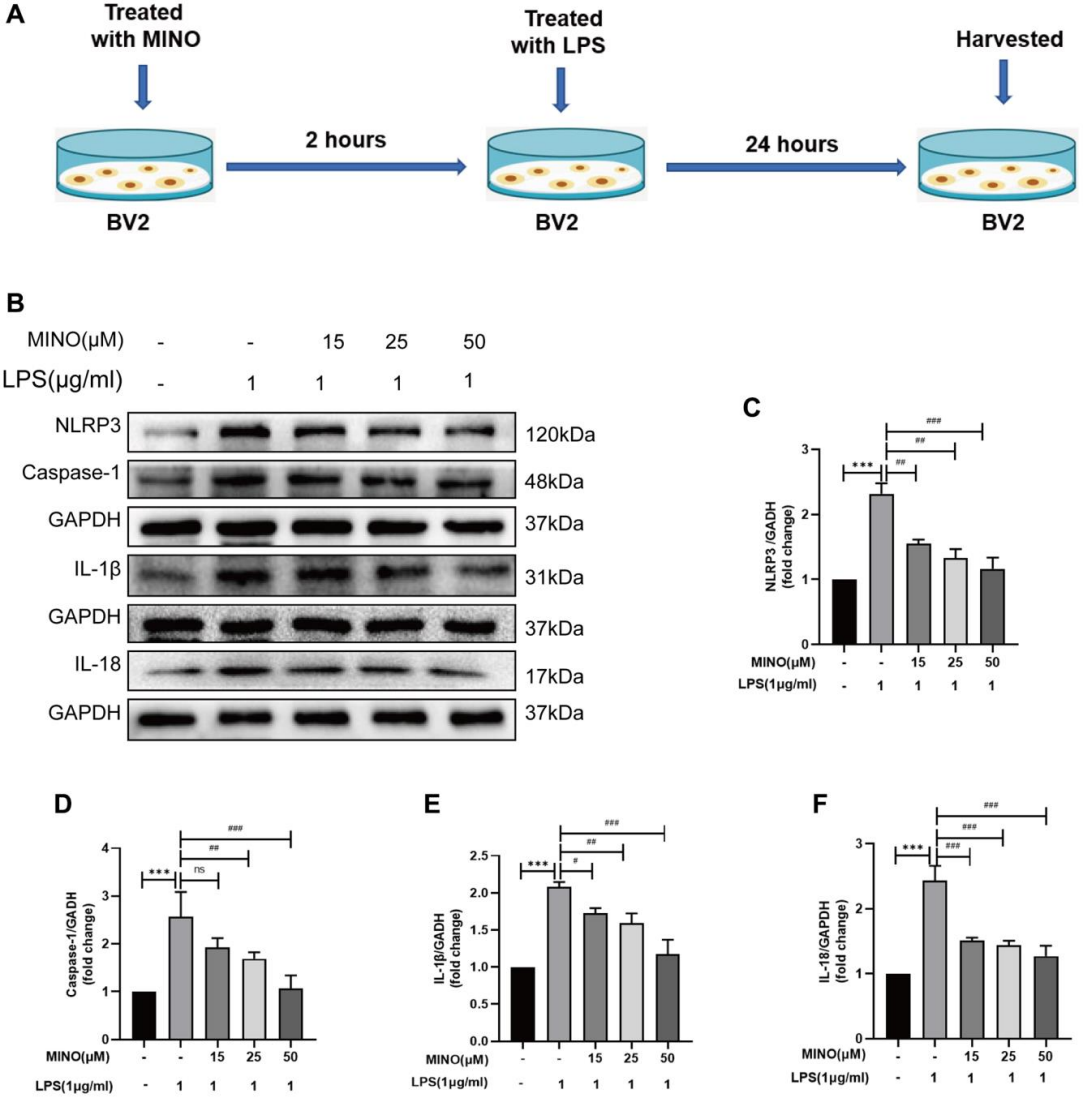
**Minocycline suppressed NLRP3/caspase-1 pathway activation in LPS-treated BV2 cells.** (**A**) The protocol of BV2 cell treatments. (**B**–**F**) The expressions of NLRP3, caspase-1, IL-1β, and IL-18 was determined by Western blotting assay. Data were presented as mean ± SD; *n* = 4; ns: statistically non-significant; ^*^P < 0.05, ^**^P < 0.01, ^***^P < 0.001 vs. control group; ^#^*P* < 0.05, ^##^*P* < 0.01, ^###^*P* < 0.001 vs. LPS group.

The treatment protocol for BV2 cells is outlined in Figure 6A. To determine the appropriate concentration of minocycline for treatment, a CCK-8 assay was performed to evaluate the viability of BV2 cells after being exposed to different concentrations of minocycline (ranging from 0–100 μM) for 24 hours. The findings revealed that minocycline treatment up to 50 μM did not have any harmful effects on BV2 cells, as demonstrated in Figure 5A.

In accordance with earlier investigations [35, 36], BV2 cells were subjected to 1 μg/ml of LPS for the purpose of stimulating them and establishing an *in vitro* model of neuroinflammation. Subsequently, the effect of pretreating BV2 cells with minocycline on cell viability after LPS stimulation was evaluated. The outcomes obtained from the CCK-8 assay exhibited a noteworthy enhancement in BV2 cell viability upon minocycline pretreatment following LPS stimulation, and this effect was found to be dose-dependent, as illustrated in Figure 5B.

Previous research has shown that apoptosis occurs in BV2 cells when treated with 1 μg/ml LPS [37, 38]. To determine the impact of minocycline on LPS-induced apoptosis in BV2 cells, we utilized Hoechst 33342/PI staining. The outcomes demonstrated a significant rise in the number of BV2 cells undergoing apoptosis in the LPS group compared to the control group. Importantly, pretreatment with minocycline dose-dependently decreased the number of apoptotic BV2 cells in the LPS+MINO group in comparison to the LPS group, as shown in Figure 5C, 5D. Overall, these findings indicate that minocycline possesses a protective effect on BV2 cells.

### Minocycline suppressed NLRP3/Casapase-1 pathway activation in LPS-treated BV2 cells

The role of the inflammatory response triggered by NLRP3 in LPS-induced neurotoxicity is crucial [39, 40]. In this study, we investigated the potential of minocycline to inhibit NLRP3-triggered inflammatory responses in a laboratory setting. Figure 6B–6F illustrates that the levels of NLRP3, caspase-1, IL-1β, and IL-18 proteins were higher in the LPS group compared to the control group. However, pretreatment with minocycline effectively suppressed the expression of these proteins, as demonstrated by the LPS + MINO group. Consequently, treatment with minocycline alleviated the activation of the NLRP3/caspase-1 pathway in response to LPS *in vitro*.

### Activation of NLRP3 reverses the protective effect of minocycline *in vitro*

**Figure 7:**
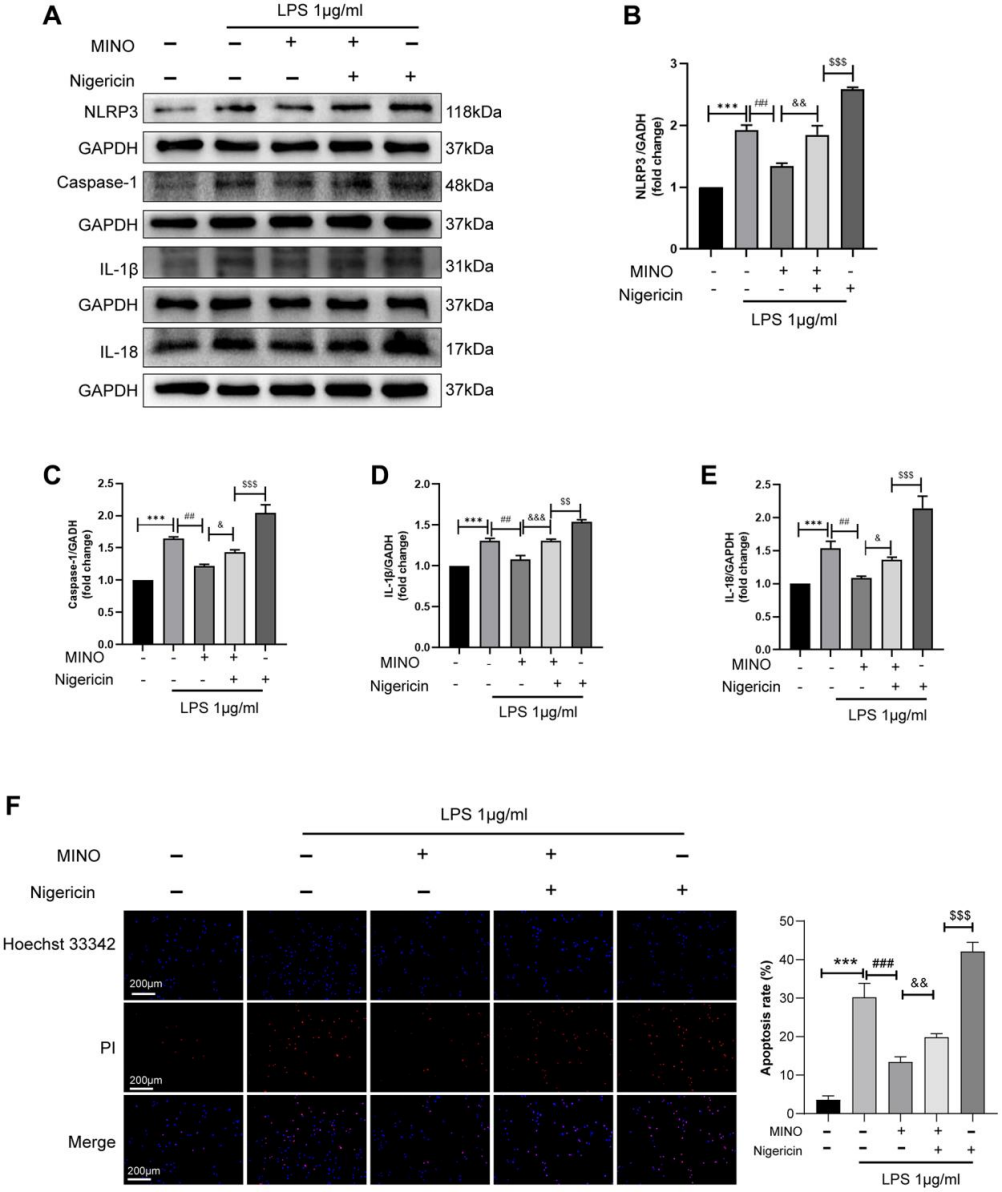
**Activation of NLRP3 reverses the protective effect of minocycline *in vitro*.** (**A**–**E**) The expression of NLRP3, caspase-1, IL-1β, and IL-18 was determined by Western blotting assay. (**F**) The apoptosis of BV2 cells was detected by the Hoechst 33,342/PI staining assay. Data were presented as mean ± SD; *n* = 4; ^*^p < 0.05, ^**^p < 0.01, ^***^p < 0.001 vs. control group; ^#^*p* < 0.05, ^##^*p* < 0.01, ^###^*p* < 0.001 vs. LPS group; ^&^*p* < 0.05, ^&&^*p* < 0.01, ^&&&^*p* < 0.001 vs. MINO + LPS group; ^$^*p* < 0.05, ^$$^*p* < 0.01, ^$$$^*p* < 0.001 vs. MINO + Nig + LPS group.

In order to examine the hypothesis that MINO enhances cognitive impairment by inhibiting NLRP3 expression, the combination of nigericin and MINO was administered. As illustrated in (Figure 7A–7E), the coadministration of MINO and LPS effectively suppressed the activation of NLRP3, caspase-1, IL-1β, and IL-18 proteins. However, the expression levels of these proteins were restored by the introduction of nigericin. Furthermore, the utilization of Hoechst 33342/PI staining unveiled that MINO hindered LPS-induced BV2 cell apoptosis, whereas the protective effect of MINO on BV2 cell survival was reversed by nigericin (Figure 7F). In brief, these findings demonstrate that MINO exerts neuroprotective properties by impeding NLRP3 activation.

## DISCUSSION

**Figure 8:**
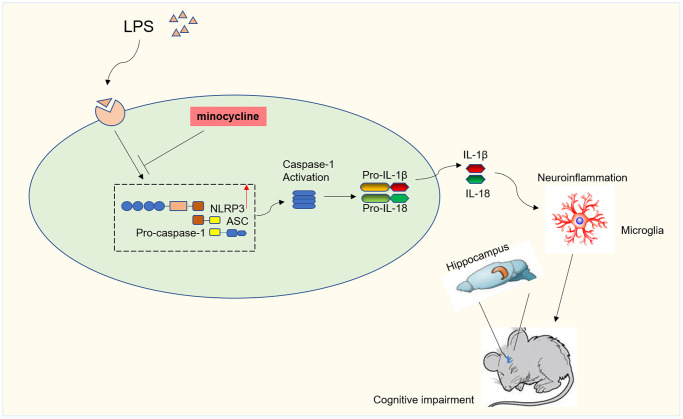
**Diagram illustrating the major findings of this study.** The results showed minocycline treatment could improve LPS-stimulated cognitive dysfunction by inhibiting NLRP3/caspase-1 pathway.

In the current investigation, we have successfully illustrated the substantial alleviation of LPS-induced cognitive dysfunction through the administration of MINO. This remarkable cognitive improvement is attributed to the potent anti-inflammatory and anti-apoptotic effects of MINO. Specifically, MINO effectively rectified the LPS-induced cognitive impairment by effectively suppressing the NLRP3/caspase-1 signaling. To evaluate the underlying mechanisms, we conducted experiments involving pretreatment of both mice and BV2 microglia with MINO. The outcomes of these experiments revealed that LPS significantly upregulated the expression of NLRP3/caspase-1 and proinflammatory cytokines, specifically IL-1β and IL-18. Remarkably, our study also discovered that pretreatment with MINO resulted in the inhibition of neuroinflammation, suppression of microglial activation, and downregulation of NLRP3/caspase-1 expression. Moreover, this intervention successfully restored the learning and memory abilities in LPS-treated mice. In light of these findings, our study provides conclusive evidence that MINO effectively mitigates LPS-induced cognitive dysfunction by effectively suppressing the NLRP3/caspase-1 signaling pathway (refer to Figure 8 for visual representation).

Cognitive impairment is primarily caused by the phenomenon of neuroinflammation. To investigate the underlying mechanisms of both neurodegenerative diseases and cognitive impairment, researchers commonly employ LPS as a means to construct models of neuroinflammation [5, 6]. In a previous study, we were able to illustrate that neuroinflammation induced by LPS leads to cognitive dysfunction. Both the MWM and the Y-maze tests were utilized to evaluate this cognitive decline [38]. By utilizing the same approach, our current study also verified that LPS induces cognitive decline, thus confirming the success of the LPS-induced cognitive dysfunction model. Minocycline, a powerful antibiotic with broad-spectrum effects, demonstrates the ability to permeate the blood-brain barrier and effectively distribute within the brain. Consequently, it effectively suppresses the activation of microglia [41, 42].

The study revealed that pretreatment with MINO had the effect of reducing the expression of Iba-1, which is a marker for microglial activation. This was confirmed through the use of IHC and WB blotting. Additionally, the results of the MWM test indicated that MINO pretreatment was able to improve cognitive impairment that had been induced by LPS. These findings clearly demonstrate that MINO has the ability to inhibit microglial activation and enhance cognitive function.

The NLRP3 inflammasome plays a vital role in the innate immune system of the body, and its activation results in the maturation of caspase-1 and the secretion of inflammatory cytokines, namely IL-1β and IL-18. These cytokines subsequently trigger cell apoptosis [43]. Hence, the suppression of NLRP3 inflammasome activation is of great significance in treating inflammatory diseases. Earlier studies have shown that MINO can effectively prevent brain damage caused by cerebral hemorrhage or ischemia by inhibiting the activation of the NLRP3 inflammasome [44, 45].

Our findings indicate that pretreatment with MINO leads to a decrease in the expression of NLRP3/caspase-1 and inflammatory cytokines, both *in vivo* and *in vitro*. Furthermore, MINO pretreatment mitigated the damage to hippocampal tissue and the occurrence of apoptosis in BV2 cells induced by LPS. These findings provide evidence that MINO enhances cognitive function impairment caused by LPS, potentially by inhibiting the NLRP3/caspase-1 pathway.

We additionally investigated if the cognitive function enhancement provided by MINO occurs through the inhibition of the pathway involving NLRP3 and caspase-1. In BV2 cells, we combined MINO with Nigericin, a specific activator of NLRP3. Previous research conducted by Zhang et al. demonstrated that the activation of NLRP3 by Nigericin can reverse the protective benefits of Calycosin on H9C2 cells [46]. Our study yielded results indicating that Nigericin is capable of significantly increasing the expression levels of NLRP3, caspase-1, IL-1β, and IL-18 in BV2 cells, thereby inducing cellular apoptosis. Nevertheless, MINO effectively inhibits the induction caused by Nigericin. These findings strongly suggest that MINO’s inhibition of the NLRP3/caspase-1 pathway contributes to the alleviation of LPS-induced cognitive impairment.

Our findings also revealed the harm to neurons caused by LPS. Previous research has indicated that this effect may be influenced by microglia [47, 48]. Microglia, which are essential immune cells in the central nervous system (CNS), play a crucial role not only in maintaining immune balance but also in brain development and cognitive function. Consequently, disruptions in microglial function are strongly linked to various disorders, including cognitive decline and Alzheimer’s disease [49]. Witcher et al. conducted a study comparing the impact of diffuse traumatic brain injury on neurons in the presence and absence of microglia, as well as microglia-mediated inflammation. These findings demonstrated that without microglia, neurons did not experience any traumatic brain injury-induced changes in gene expression or structure. This implies that microglia have a crucial role in promoting persistent neuropathology and long-term impairment of neuronal homeostasis following traumatic brain injury [50].

### Limitations

The present study has certain limitations. Firstly, this investigation solely focused on evaluating the inhibitory effects of minocycline on microglial activation and did not take into account its impact on other central nervous system (CNS) cell types, such as astrocytes. Secondly, the study exclusively utilized nigericin as the activator of NLRP3 and did not investigate the potential interaction between MINO and NLRP3 via enforced NLRP3 overexpression. Thirdly, we did not provide a comprehensive elucidation of the mechanisms by which LPS in microglia induces neuronal damage. Lastly, our experimental and treatment approaches primarily relied on BV2 cells to confirm our findings. Nevertheless, it is important to highlight that the validation of our research could be strengthened by incorporating other relevant cell lines, including astrocytes and oligodendrocytes. By integrating these additional cell lines, a more holistic understanding of the anti-inflammatory properties of MINO in the context of neuroinflammation can be achieved. Consequently, further research is warranted to explore the supplementary functionalities of minocycline within the CNS.

## CONCLUSION

The results of this investigation demonstrated that the prior administration of minocycline effectively mitigated cognitive dysfunction induced by LPS through the inhibition of the pathway involving NLRP3 and caspase-1. These discoveries substantiate the potential of minocycline as an innovative therapeutic strategy for addressing impairments in cognitive function.

## Supplementary Materials

Supplementary Figure 1
