# Supplementary material for: Minocycline alleviates LPS-induced cognitive dysfunction in mice by inhibiting the NLRP3/caspase-1 pathway
Source: Aging (Albany NY). 2024 Feb 6;16(3):2989–3006. doi: 10.18632/aging.205528 (PMC10911373; doi:10.18632/aging.205528)
Supplement: Supplementary Figure 1 [file aging-16-205528-s001.pdf]

SUPPLEMENTARY FIGURE

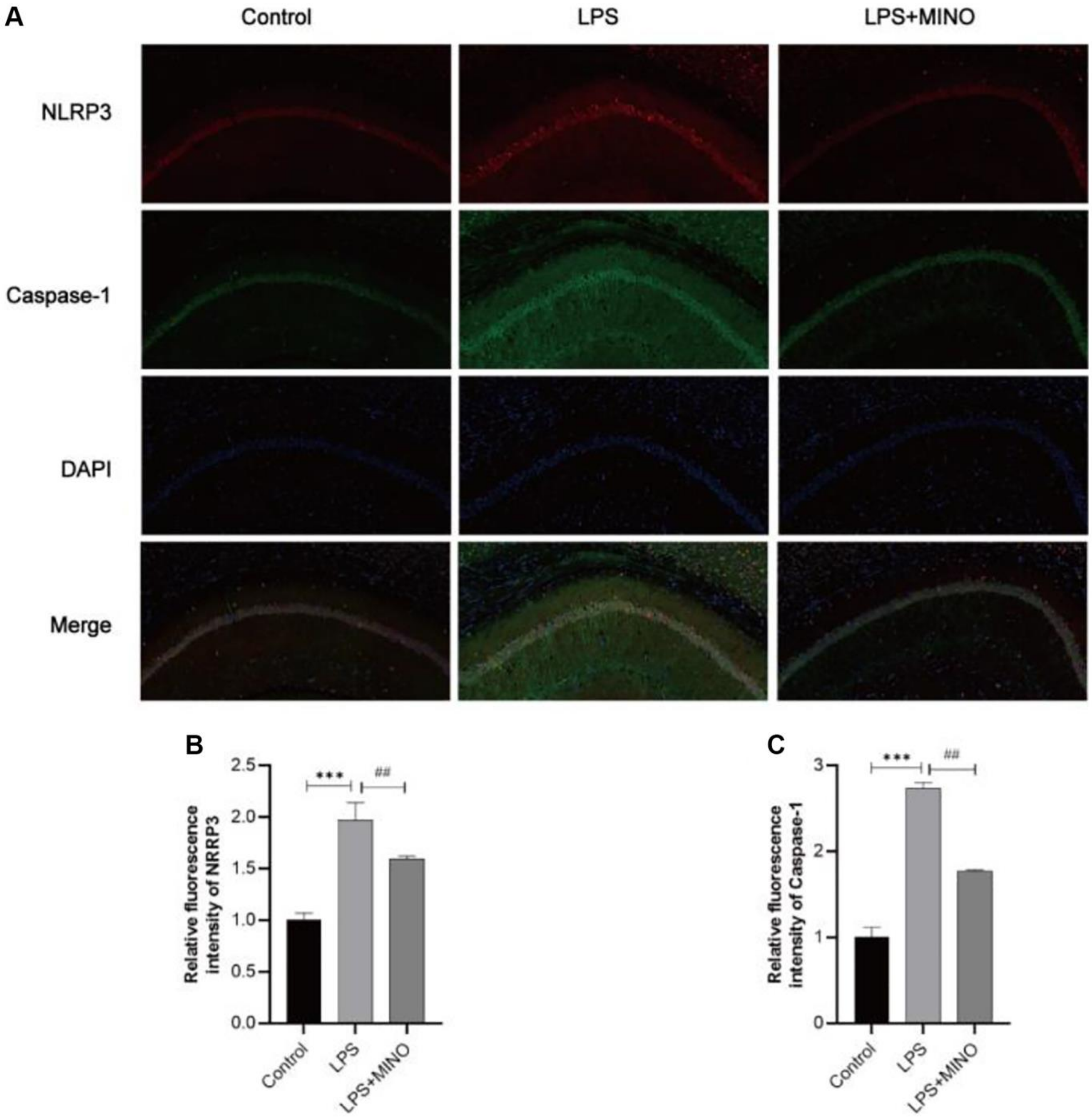

Supplementary Figure 1. Minocycline inhibited LPS-induced microglial activation in the mice hippocampus. (**A**) An immunofluorescence staining assay was performed to detect the expression level of NLRP3 and Caspase-1 in the mice hippocampus, and (**B**, **C**) their statistical graphs. Data were presented as mean ± SD; *n* = 5-6 mice per group. $^{*}P < 0.05$, $^{**}P < 0.01$, $^{***}P < 0.001$ vs. Control group; $^{\#}P < 0.05$, $^{\#\#}P < 0.01$, $^{\#\#\#}P < 0.001$ vs. LPS group.
